# Insights into the posttranslational assembly of the Mo-, S- and Cu-containing cluster in the active site of CO dehydrogenase of *Oligotropha carboxidovorans*

**DOI:** 10.1007/s00775-014-1201-y

**Published:** 2014-11-07

**Authors:** Astrid M. Pelzmann, Frank Mickoleit, Ortwin Meyer

**Affiliations:** Department of Microbiology, University of Bayreuth, Universitätsstrasse 30, 95440 Bayreuth, Germany

**Keywords:** Carbon monoxide dehydrogenase, Molybdoenzyme maturation, Copper, Phytase, DEAD-box protein

## Abstract

*Oligotropha carboxidovorans* is characterized by the aerobic chemolithoautotrophic utilization of CO. CO oxidation by CO dehydrogenase proceeds at a unique bimetallic [CuSMoO_2_] cluster which matures posttranslationally while integrated into the completely folded apoenzyme. Kanamycin insertional mutants in *coxE*, *coxF* and *coxG* were characterized with respect to growth, expression of CO dehydrogenase, and the type of metal center present. These data along with sequence information were taken to delineate a model of metal cluster assembly. Biosynthesis starts with the MgATP-dependent, reductive sulfuration of [Mo^VI^O_3_] to [Mo^V^O_2_SH] which entails the AAA+-ATPase chaperone CoxD. Then Mo^V^ is reoxidized and Cu^1+^-ion is integrated. Copper is supplied by the soluble CoxF protein which forms a complex with the membrane-bound von Willebrand protein CoxE through RGD–integrin interactions and enables the reduction of CoxF-bound Cu^2+^, employing electrons from respiration. Copper appears as Cu^2+^-phytate, is mobilized through the phytase activity of CoxF and then transferred to the CoxF putative copper-binding site. The *coxG* gene does not participate in the maturation of the bimetallic cluster. Mutants in *coxG* retained the ability to utilize CO, although at a lower growth rate. They contained a regular CO dehydrogenase with a functional catalytic site. The presence of a pleckstrin homology (PH) domain on CoxG and the observed growth rates suggest a role of the PH domain in recruiting CO dehydrogenase to the cytoplasmic membrane enabling electron transfer from the enzyme to the respiratory chain. CoxD, CoxE and CoxF combine motifs of a DEAD-box RNA helicase which would explain their mutual translation.

## Introduction

Carbon monoxide dehydrogenases (CO dehydrogenases) are enzymes which catalyze the oxidation of CO to CO_2_ yielding two electrons and two protons (CO + H_2_O → CO_2_ + 2e^−^ + 2H^+^) or the reverse reaction [1, 2]. They are key to the generation of a proton motive force across the cytoplasmic membrane for ATP synthesis or cooperate with acetyl-CoA synthase in the biosynthesis of acetyl-CoA. Two principal types of the enzyme have been identified and structurally characterized. The homodimeric CO dehydrogenase of anaerobic bacteria or archaea (e.g. *Carboxydothermus hydrogenoformans*, *Rhodospirillum rubrum* or *Moorella thermoacetica*) is an iron–sulfur protein with a [Ni-4Fe-4/5S]-cluster in the active site [3–8]. The heterohexameric CO dehydrogenase of the *α*-proteobacterium *Oligotropha carboxidovorans* and other representatives of the aerobic CO-utilizing (carboxidotrophic) bacteria is a molybdo iron–sulfur flavoprotein [9–12]. The enzyme can be grouped into the molybdenum hydroxylase (xanthine oxidase) family of Mo enzymes on the basis of its overall structural properties [9, 13]. In contrast to the mononuclear Mo enzymes and nitrogenase, CO dehydrogenase accommodates in its catalytic site a bimetallic [Mo^+VI^O_2_-S-Cu^+I^-S-Cys] cluster, which is unprecedented in nature [10, 11, 14]. The Mo ion of the cluster is coordinated by the ene-dithiolate of the molybdopterin cytosine dinucleotide cofactor (MCD) [10, 15–18]. The Cu ion is ligated by the *γ*S of the cysteine residue 388 [10] which is part of the unique signature VAYRCSFR [19, 20]. The copper in the bimetallic cluster is promiscuous to some extent since it can be substituted in vitro for silver yielding a functional enzyme [21]. From experiments with an *n*-butylisocyanide inhibitor, a thiocarbonate transition state (S-CO_2_) in the CO oxidation reaction was proposed [11]. However, quantum chemical modeling suggested that the first step in the catalytic cycle is the binding of CO via its carbon atom to the Cu^1+^-ion followed by a transfer of the equatorial oxygen-derived ligand on the Mo ion which involves the glutamate residue 763 [22]. In addition, only the bis-oxo but not the hydroxo-oxo complex oxidizes CO exothermically [23].

The assembly of the iron–molybdenum cofactor (FeMoco) of nitrogenase and the molybdenum cofactor (Moco) of non-nitrogenase molybdoenzymes is a complex process which follows different routes. In all instances, these cofactors are separately synthesized and then inserted into an apo-protein [13, 24–26]. The biosynthesis of FeMoco proceeds on a number of scaffold proteins prior to its delivery to its target location NifDK [27]. The process involves the stepwise formation of an 8Fe core and the subsequent maturation of this core upon incorporation of molybdenum and homocitrate. Specific proteins of the XdhC family are required for the maturation of enzymes of the molybdenum hydroxylase family in bacteria [25]. The suggested functions of XdhC are to (i) assist in the sulfuration of the Mo ion by interaction with the l-cysteine desulfurase NifS4, (ii) stabilize the sulfurated form of Moco before the insertion into the apoenzyme, (iii) insert the sulfurated Moco into the apoenzyme, (iv) hinder the exchange of the sulfur ligand of Moco against an oxygen atom, and (v) act as a chaperone for the proper folding of the enzyme after Moco insertion [25, 28].

The 14.54-kb *cox* gene cluster on the circular DNA megaplasmid pHCG3 of *O. carboxidovorans* OM5 includes the subcluster *coxMSL* which codes for the CO dehydrogenase polypeptides and the subcluster *coxDEFG* [19, 20]. It has been suspected that *coxDEFG* has functions in the posttranslational processing of sulfur and Cu [12]. In contrast to all other molybdoenzymes, where the Moco is assembled outside of its target proteins, sulfuration of CO dehydrogenase proceeds on the Moco in its [MoO_3_] form already incorporated into the completely folded apoenzyme [29].

In the present work, we have studied metal cluster composition, structure and function of CO dehydrogenase synthesized in mutants of *O. carboxidovorans* OM5 in which the genes *coxE*, *coxF* and *coxG* have been disrupted by insertional mutagenesis. The purified CO dehydrogenases were characterized by chemical analysis, electron paramagnetic resonance spectroscopy (EPR), in vitro reconstitution experiments and the use of sulfur compounds as probes for the accessibility of the active site and reporters of sulfur interaction with incomplete forms of the metal cluster. Furthermore, we use bioinformatic evidence to propose the hypothetical involvement of CoxD, CoxE and CoxF as DEAD-box protein RNA helicases in the translation of CoxE and CoxF. The results support a model of the posttranslational biosynthesis of the bimetallic cluster in the active site of CO dehydrogenase.

## Materials and methods

### Organisms and cultivation

*Oligotropha carboxidovorans* strain OM5 (DSM 1227, ATCC 49405; [30]) and its mutants carrying a kanamycin resistance cassette inserted into the genes *coxD*, *coxE, coxF* or *coxG* were grown in 50-liter fermentors (model Biostat, Braun Melsungen, Germany) in a mineral medium [31] at 30 °C. Cultures were supplied with gas mixtures of (% by volume): 45 CO, 5 CO_2_ and 50 air; 40 H_2_, 10 CO_2_ and 50 air; 30 H_2_, 5 CO_2_, 30 CO and 35 air. Bacteria, harvested in the late exponential growth phase, were stored frozen at −80 °C until use.

### Insertional mutagenesis of *coxE*, *coxF* and *coxG*

The gene *coxE* is part of the 14.5 kb cluster *coxBCMSLDEFGHIK* of *O. carboxidovorans* [19, 20, 32, 33]. The plasmid pETE1 is a recombinant construct of vector pET11a (Novagen, Heidelberg, Germany), which carries the fragment of *coxE*. pETE1 was isolated from *E. coli* DH5*α* and a central 0.6 kb part of the *coxE* fragment was replaced by a kanamycin resistance cassette isolated from plasmid pUC4KIXX (Amersham Pharmacia Biotech, Freiburg, Germany) from *E. coli* DH5α [34]. The recombinant plasmid is referred to as pETEkm. The fragment of *coxE* was isolated from pETEkm and cloned into the suicide plasmid pSUP 201-1 isolated from *E. coli* S17-1 [35]. The resulting plasmid pSUPEkm2 was transformed into *E. coli* S17-1. Finally, *coxEkm* was transferred from *E.* *coli* S17-1 to *O. carboxidovorans* OM5 by conjugation. Successful recombination was checked by Southern blotting employing a KIXX probe, which was directed against the kanamycin resistance cassette and a specific probe for *coxE*. For the KIXX probe the 1.2 kb KIXX fragment from the pUC4KIXX was isolated and labeled with digoxigenin. For the specific *coxE* probe, the plasmid pCAC1 [19], which carries an *Eco*RV fragment of pHCG3 with the *coxE* fragment, was isolated from *E.* *coli* DH5α. The sequence was amplified using PCR, and purity was checked by agarose gel electrophoresis. The PCR product was hydrolyzed with *Msc*I, and the resulting fragments were separated by agarose gel electrophoresis. A 0.75 kb fragment was eluted from the gel and labeled with digoxigenin. The plasmid DNA of the *O. carboxidovorans* wild type and mutant was isolated and restricted with *Eco*RV and *Hind*III. Agarose gel electrophoresis was used to separate the fragments. After transferring the fragments onto a nylon membrane, digoxigenin-labeled probes were added and detection was performed. A 3.2 kb *Eco*RV fragment of pETEkm was used as a positive control.

For the insertional mutagenesis of *coxF* the corresponding fragment was cloned into the vector pET11a (Novagen, Heidelberg, Germany) resulting in the recombinant construct pETFG1, which could be isolated from *E. coli* DH5α. For in vitro mutagenesis of the *coxF* gene, a kanamycin resistance cassette was isolated from plasmid pUC4KIXX (Amersham Pharmacia Biotech, Freiburg, Germany) of *E. coli* DH5α [34], inserted into the *coxF* fragment and transformed into *E. coli* DH5α. The recombinant plasmid is referred to as pETFG1 km. The *coxFkm* fragment isolated from pETFG1 km was cloned into the suicide plasmid pSUP 201-1 isolated from *E. coli* S17-1 [35], resulting in the plasmid pSUPFkm. After the transformation of pSUPFkm into *E. coli* S17-1, the *coxFkm* construct was transferred from *E. coli* S17-1 to *O.* *carboxidovorans* OM5 by conjugation. Mutagenesis of the *coxF* gene could be confirmed by Southern Blotting employing a KIXX probe directed against the inserted kanamycin resistance cassette. This probe was produced by isolating the 1.2 kb KIXX fragment from the plasmid pUC4KIXX followed by labeling with digoxigenin. Insertional mutagenesis of *coxG* followed the same procedure as described for *coxF*.

### Translational analysis

For translational analyses, cell-free extracts of wild type and the mutants in *coxD* (D::km), *coxE* (E::km) and *coxF* (F::km) were separated by denaturing PAGE [36]. Protein bands were blotted onto polyvinylidene fluoride (Roth, Karlsruhe, Germany) and used for immunodetection with IgG antibodies (rabbit) directed against the polypeptides CoxD, CoxE and CoxF (Eurogentec, Seraing, Belgium).

### Enzyme purification and activity assay

Bacteria suspended in 50 mM KH_2_PO_4_/KOH (pH 7.2) were disintegrated with a French-pressure cell at maximum pressure (American Instruments Company, Silverspring, Maryland, USA). The resulting extracts were subjected to ultracentrifugation (2.5 h at 100,000×*g*, Centrikon T4510, Kontron, Eching, Germany). Purifications of CO dehydrogenase started from soluble supernatants involving anion exchange chromatography, hydrophobic interaction chromatography, gel filtration and chromatography on hydroxylapatite [10, 14, 29]. Purity was checked by native PAGE (7.5 % acrylamide, 50 mM Tris/384 mM glycine, pH 8.5) stained for protein with Coomassie Brilliant Blue 250. The amount of protein in CO dehydrogenase bands appearing on gels was quantitated by video densitometry (ImageJ; http://rsb.info.nih.gov/ij/). Purified preparations of CO dehydrogenase were frozen in liquid nitrogen and stored at −80 °C until use. CO-oxidizing activity was assayed photometrically employing INT [1-phenyl-2-(4-iodo-phenyl)-3-(4-nitrophenyl)-2H-tetrazolium chloride] as electron acceptor [37] or analyzed by activity staining on native PAGE. One unit of CO dehydrogenase activity is defined as 1 µmol of CO oxidized per min at 30 °C. Protein estimation followed published procedures [38, 39]. Amounts of purified CO dehydrogenase were also determined from the visible absorption spectrum at 450 nm employing an extinction coefficient (*ε*_450_) of 72 mM^−1^ cm^−1^ at 450 nm [40] and a molecular mass of 277,074.37 Da. Ultraviolet/visible spectra were recorded on a spectrophotometer (BioMate 6, Thermo-scientific, Madison WI, USA). Functionalities were determined as follows: The absorption differences at 450 nm of CO dehydrogenase in the air-oxidized state minus the dithionite-reduced state or minus the CO-reduced were determined. The percentage of the latter relative to the former was taken as functionality. Functionality describes the portion of catalytically active CO dehydrogenase in a preparation which also contains inactive enzyme species. The method for the determination of functionality is based on the fact that CO oxidation at the catalytic site releases two electrons which travel to the FAD cofactor, via the iron–sulfur centers. Bleaching of the flavin occurs only in catalytically active enzyme species and not in inactive enzyme species because CO is not oxidized and consequently electrons are not released. Functionality was determined as follows: Enzyme solution contained in a serum-stoppered cuvette was sparged with pure CO for 30 min at room temperature. Subsequently visible spectra were recorded with time until the decrease in absorption at 450 nm came to a standstill (this usually took less than 45 min). Then another sample of the same enzyme in a serum-stoppered cuvette was saturated with pure N_2_, and sodium dithionite was injected through the septum to achieve 5 mM final concentration. Again, spectra were recorded until the absorption decrease at 450 nm went to completion (this usually took about 5 min). The quantity of the absorption decrease at 450 nm of the dithionite-reduced enzyme reflects the total flavin content and measures the amount of active and inactive enzyme present. It was set 100 %. The quantity of the absorption decrease at 450 nm obtained in the presence of CO indicates the amount of CO dehydrogenase species capable of oxidizing CO and reducing the FAD cofactor contained in the enzyme. The term “functionality” refers to the percentage of the extent of reduction at 450 nm achieved with CO relative to the reduction obtained with dithionite. Other than the specific activity, which describes µmol CO oxidized min^−1^ mg^−1^, functionality of CO dehydrogenase preparations indicates the amount of enzyme species capable of oxidizing CO relative to the sum of catalytically active and inactive species.

### Determination of sulfane sulfur, FAD and metals

Sulfane sulfur was determined by treating samples of CO dehydrogenase with potassium cyanide followed by colorimetric analysis of the resulting thiocyanate as FeSCN [12]. FAD was determined from its absorption at 450 nm in supernatants of trichloroacetic acid precipitates of CO dehydrogenase, neutralized with 2.4 mM K_2_HPO_4_ [9]. Copper, molybdenum and zinc in CO dehydrogenase were determined by flame atomic absorption spectroscopy (model 1100 B, Perkin Elmer, Überlingen, Germany).

### Reconstitution of apo-CO dehydrogenase

CO dehydrogenases in their as isolated state (5 mg ml^−1^ in 50 mM HEPES, pH 7.2) were treated under anoxic conditions with 150 µM Cu^1+^-(thiourea)_3_ or with sodium sulfide and sodium dithionite (5 mM each) first, followed by 150 µM Cu^1+^-(thiourea)_3_. Assays treated with sodium sulfide and sodium dithionite were incubated in the dark at 37 °C for 10 h. Assays treated with Cu^1+^-(thiourea)_3_ were incubated at 37 °C for 2–4 h in the dark. For the removal of Cu and sulfane sulfur, enzyme (~10 mg ml^−1^) was incubated with 5 mM potassium cyanide for 24 h and then, gel filtered on PD10 ready-to-use columns (Sephadex G25, GE Healthcare, Little Chalfont, UK). Finally, CO dehydrogenase in the excluded volume was treated with sulfide/dithionite and copper as above. The procedures have been adopted from [41].

### Electron paramagnetic resonance (EPR) spectroscopy

X-band EPR spectra were recorded on a Bruker EMX spectrometer equipped with an ESR 900 helium cryostat (Oxford Instruments, Oxon, UK) as described [14]. Spectra were recorded at 120 K applying a microwave frequency of 9.47 GHz, 1 mT modulation amplitude and 10 mW microwave power. The magnetic field was calibrated with a diphenylpicrylhydrazin sample. Assays containing CO dehydrogenase (12 mg ml^−1^ in 50 mM HEPES, pH 7.2) were made anoxic by sparging with N_2_ and then reduced with 5 mM sodium dithionite or sparged with pure CO for 30 min and frozen in liquid N_2_. Where indicated, assays were amended with 15 mM cysteine, 15 µM Na_2_S or 15 mM 2-mercaptoethanol and kept at room temperature for 25 min. Samples were kept in liquid nitrogen until use.

### Miscellaneous methods and chemicals

All chemicals employed were of analytical grade and purchased from the usual commercial sources. Bioinformatic tools employed: The online server SABLE (Solvent AccessiBiLiEs of amino acid residues in proteins and improved prediction of secondary structures http://sable.cchmc.org/; [42]) for secondary structure predictions; the Conserved Domain Database (http://www.ncbi.nlm.nih.gov/Structure/cdd/wrpsb.cgi; [43]) for domain search on Cox proteins; T-Coffee (http://www.tcoffee.org/) for structural sequence alignments of CoxG.

## Results

### Bioinformatic characterization of the proteins CoxDEFG

**Fig. 1:**
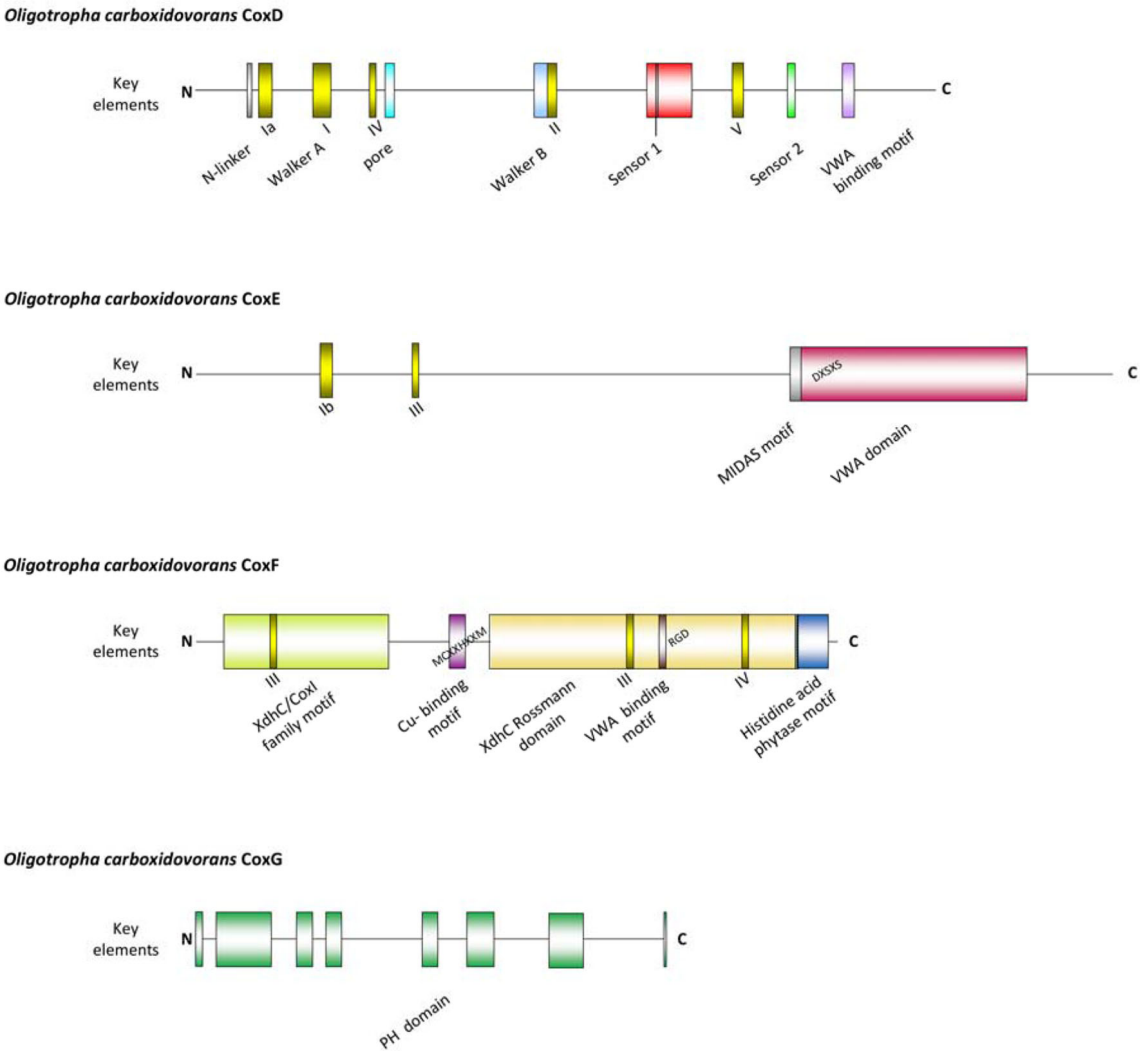
Motifs on the amino acid sequences of the polypeptides CoxD, CoxE, CoxF and CoxG of *O. carboxidovorans* OM5. For bioinformatic programs employed refer to the methods section. Color coding: DEAD-box protein motifs, *yellow*; PH domain elements, *green*; VWA domain, *red*; histidine acid phytase motif, *blue*. *MIDAS* metal ion adhesion site, *PH domain* pleckstrin homology domain, *VWA* von willebrand factor A (integrin I), *XdhC* xanthine dehydrogenase C

The CoxD protein is a novel AAA+ ATPase (Fig. 1) which has the key elements of an AAA+ domain in the same arrangement and same positions as in the BchI component of Mg^2+^-chelatase [29]. CoxD operates in the maturation of the CO dehydrogenase bimetallic cluster, particularly in the sulfuration of the [MoO_3_]-site and in ATP-dependent chaperone functions [29, 44]. In addition to the AAA-signatures, the CoxD sequence reveals five homologues of conserved motifs suggestive of the DEAD-box protein family of RNA helicases (Fig. 1). DEAD-box proteins have been shown to support the maturation of RNA molecules and to be required for translation initiation [45]. CoxE (399 aa; 44,235 Da; seventeen predicted *α*-helices and six *β*-sheets) is remarkably high in arginine (12.5 %, [19]) and contains an integrin I domain at its C-terminus (Fig. 1). Von willebrand proteins function in processes such as cell adhesion or multiprotein complex formation [46]. Furthermore, CoxE also carries two DEAD-box protein motifs (Fig. 1). CoxF (280 aa; 29,346 Da; nine *α*-helices and ten *β*-sheets) reveals an XdhC protein family motif at its N-terminus in combination with an XdhC Rossman domain near the C-terminus, a putative Cu-binding motif [47], an integrin-binding motif [46], a histidine acid phytase motif [48] at the C-terminus, and two scattered DEAD-box motifs (Fig. 1). Phytases (*myo*-inositol hexakisphosphate phosphohydrolases) from fungi, bacteria, yeasts or plants catalyze the partial or complete hydrolytic removal of orthophosphates from phytates (*myo*-inositol hexakisphosphates) [48]. Phytic acid is a potent chelator of divalent cations [48] with the order of affinity Cu^2+^ ≥ Zn^2+^ > Mn^2+^ > Mg^2+^ > Co^2+^ > Ni^2+^ [49]. The release of phosphate groups from phytate by phytase results in the release of metal ions [48]. CoxG (205 aa; 21,559 Da; six predicted *α*-helices and eight *β*-sheets) contains a PH domain (pleckstrin homology domain) (Fig. 1). The PH domain encompasses about 100 loosely conserved amino acids and is found in numerous different types of mostly eukaryotic proteins with functions in intracellular signaling, cellular membrane dynamics and the cytoskeleton [50, 51].

PH domains can bind phosphatidylinositol membrane lipids, membrane proteins or both. They determine the membrane localization of the proteins in which they reside, thus presenting them to required cellular compartments or enabling them to interact with other components of the signal transduction pathways.

The functions of the genes *coxE* and *coxF* are essential for the utilization of CO by *O. carboxidovorans*, whereas *coxG* is not required.

**Fig. 2:**
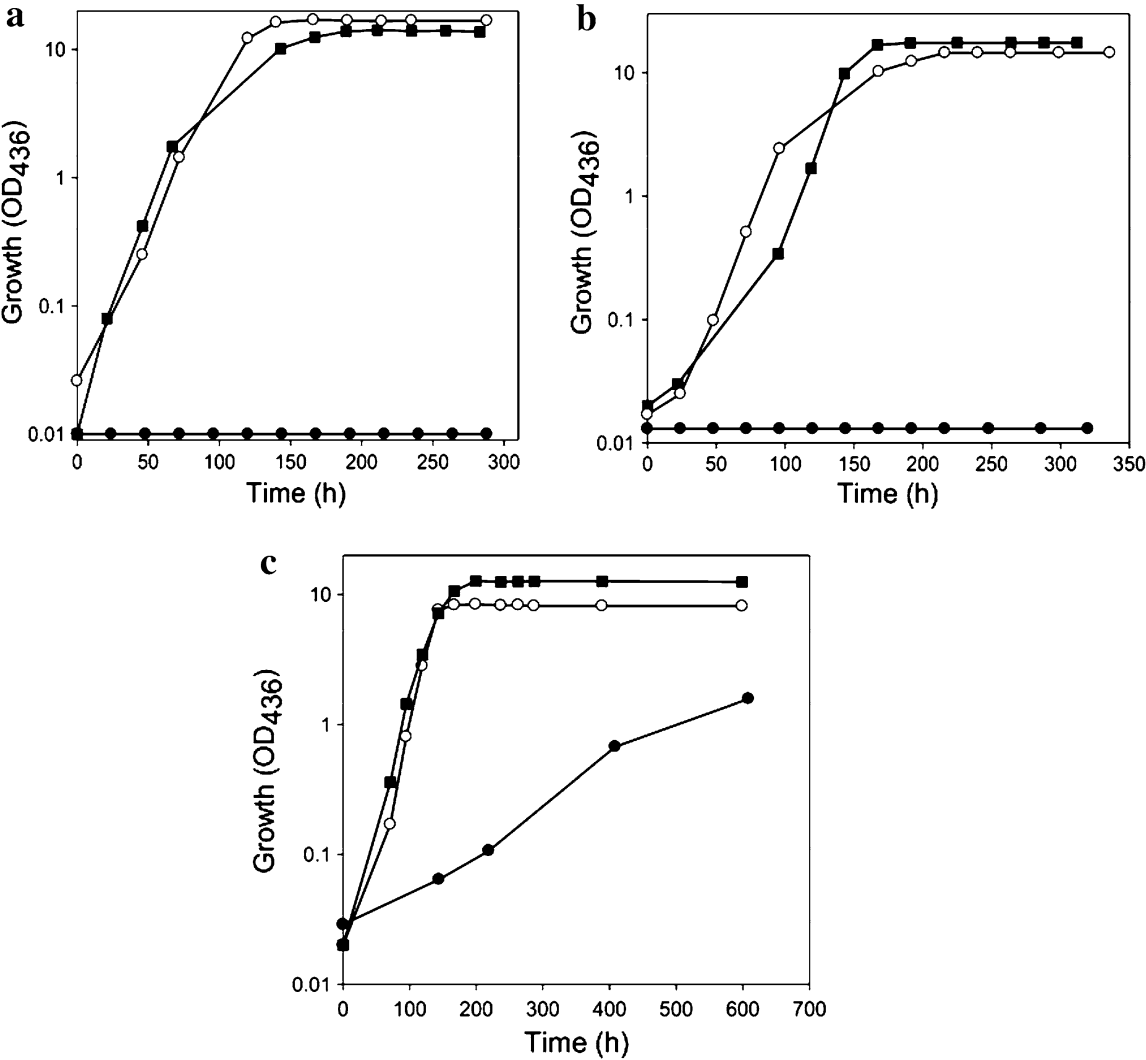
Growth experiments with strains of *O. carboxidovorans* in which the genes *coxE* (E::km) (**a**), *coxF* (F::km) (**b**) or *coxG* (G::km) (**c**) were inactivated by insertional mutagenesis. Bacteria were cultivated under chemolithoautotrophic conditions employing the following gas mixtures (v/v): 45 % CO, 5 % CO_2_, and 50 % air (*filled circle*); 40 % H_2_, 10 % CO_2_, and 50 % air (*open circle*); and 30 % H_2_, 5 % CO_2_, 30 % CO, and 35 % air (*filled square*). Each data point represents the average of three optical density measurements on separate samples of the same culture. The standard deviation of such measurements was below 2.8 %. For further details see the “Materials and methods” section

In a previous paper, the D::km mutant has been studied [29]. Disruption of *coxD* led to a phenotype of D::km which was impaired in the utilization of CO, whereas the utilization of H_2_ plus CO_2_ was not affected. Under appropriate induction conditions, bacteria synthesized a fully assembled apo-CO dehydrogenase, which could not oxidize CO. Apo-CO dehydrogenase contained a [MoO_3_] site in place of the [CuSMoO_2_] cluster. Employing sodium sulfide first and then the Cu^1+^-(thiourea)_3_ complex, the non-catalytic [MoO_3_] site could be reconstituted in vitro to a [CuSMoO_2_] cluster capable of oxidizing CO. Sequence information suggested that CoxD is a MoxR-like AAA+ ATPase chaperone related to the hexameric, ring-shaped BchI component of Mg^2+^-chelatases. Similar to D::km, the mutants E::km and F::km also had lost the ability to utilize CO as a sole source of carbon and energy under aerobic chemolithoautotrophic conditions (Fig. 2a, b). This shows that the genes *coxE* and *coxF* are both obligatory for the utilization of CO as a growth substrate. As will be shown later in this paper the genes are functional in the maturation of CO dehydrogenase which is the enzyme catalyzing the oxidation of CO [1, 31]. On the other hand, the chemolithoautotrophic utilization of H_2_ plus CO_2_ by both mutant strains was not impaired, neither in the absence nor in the presence of CO (Fig. 2a, b). This is because H_2_ is oxidized by a NiFe-hydrogenase [34]. The ability to grow on H_2_ plus CO_2_ also indicates that ribulose-1,5-bisphosphate carboxylase/oxygenase (rubisco) and all other enzymes of the Calvin–Benson–Bassham (CBB) cycle employed for autotrophic CO_2_ fixation are functional in the mutants. The deletion of *coxG* led to a phenotype which was still able to utilize CO, although the generation time increased considerably from 21 h (wild type) to 149 h (G::km) (Fig. 2c).

Our hypothesis is that the sevenfold increase in generation time is caused by limited interaction of CO dehydrogenase with the cytoplasmic membrane controlled by diffusion. Compared to exponential wild-type bacteria, which contain about 50 % of the CO dehydrogenase pool at the inner aspect of the cytoplasmic membrane [52], immunocytochemical localization revealed most of the enzyme in the cytoplasm ([20], unpublished data).

However, contact of CO dehydrogenase with the membrane is required for electron transfer to drive energy transduction by the respiratory chain. This points to a role of CoxG in recruiting CO dehydrogenase to the membrane, which is further corroborated by the PH domain present on CoxG. The utilization of H_2_ and CO_2_ was not affected in the mutant G::km.

Under inducing growth conditions (i.e. H_2_ plus CO_2_ in the presence of CO) the bacteria utilize H_2_ as an energy source employing hydrogenase and CO_2_ as a carbon source employing the CBB cycle [1, 31]. The function of CO is to induce the transcription of the *cox* gene cluster on the plasmid pHCG3 [20]. Under these conditions, the mutant D::km synthesized an apo-CO dehydrogenase in which the [CuSMoO_2_] cluster was replaced by a [MoO_3_] site [29]. It was, therefore, tempting to assume that under same conditions the mutants E::km and F::km also would be able to synthesize an inactive apo-CO dehydrogenase, however, with a metal cluster representing a different stage in cluster biosynthesis. The fact that the mutant G::km was able to utilize CO under chemolithoautotrophic conditions is indicative for the presence of a catalytically active and thus fully assembled enzyme.

### Possible roles of the proteins CoxD, CoxE and CoxF in translation

**Fig. 3:**
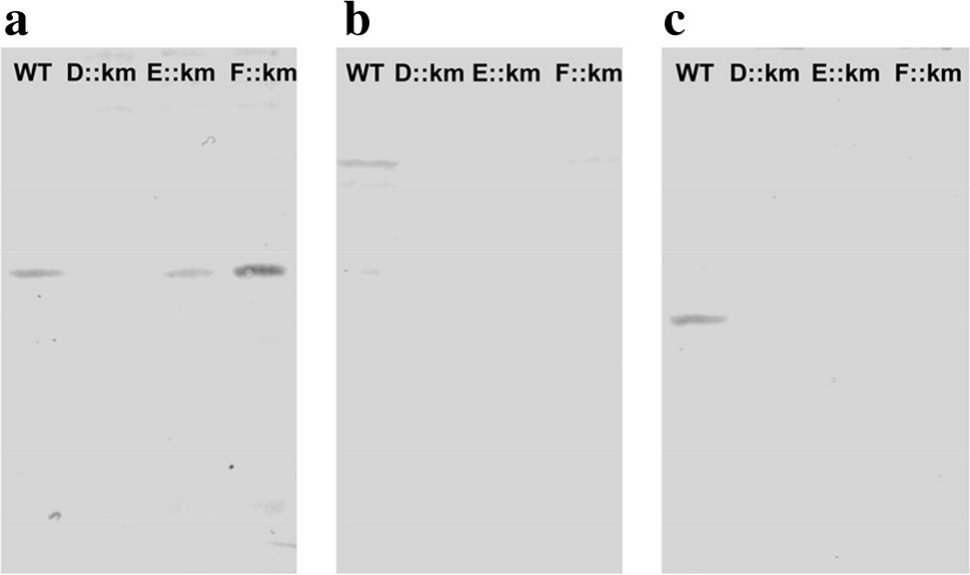
Translation of the proteins CoxD, CoxE and CoxF in cell-free crude extracts of wild-type *O. carboxidovorans* and its insertional mutants D::km, E::km or F::km. Bacteria were cultivated under chemolithoautotrophic conditions with a gas atmosphere composed of (v/v) 30 % H_2_, 5 % CO_2_, 30 % CO, and 35 % air. Cell-free crude extracts (200 µg protein/lane) were subjected to denaturing PAGE followed by Western blotting employing IgG antibodies directed against CoxD (**a**) CoxE (**b**) or CoxF (**c**)

The *cox* genes in *O. carboxidovorans* are specifically and co-ordinately transcribed under chemolithoautotrophic conditions in the presence of CO to induce the transcription of the *cox* gene cluster [19]. A probable polar effect of the km cassette insertion on *coxD* [29] or *coxE* (unpublished data) could be excluded because all neighboring *cox* genes were readily transcribed. As a polar mutation only affects expression of downstream genes, we also exclude a polar effect of km insertion into *coxF* on *coxE*. Therefore, the absence of the proteins CoxE and CoxF in the *coxD* mutant, of CoxF in the *coxE* mutant and of CoxE in the *coxF* mutant was entirely unexpected (Fig. 3). Apparently, CoxD is required for the translation of CoxE and CoxF, whereas CoxE is required for the translation of CoxF and vice versa (Fig. 3).

### Overall properties of CO dehydrogenases purified from the mutants E::km, F::km or G::km

**Fig. 4:**
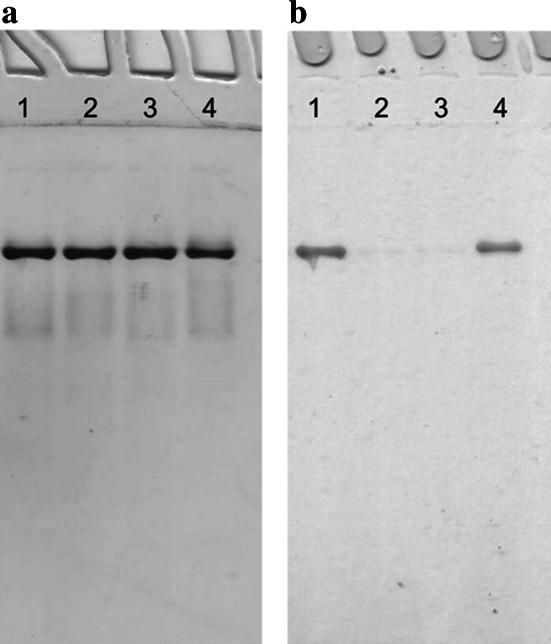
Native PAGE of CO dehydrogenases purified from *O. carboxidovorans* wild type or from insertional mutants. Lanes numbered 1 to 4 each received 30 μg of CO dehydrogenase from wild-type bacteria, or the mutants E::km, F::km or G::km, respectively. Gels were stained for protein with Coomassie Brilliant Blue (**a**) or for CO-oxidizing activity employing CO as electron donor and INT as electron acceptor (**b**). For experimental details refer to “Materials and methods”

*O. carboxidovorans* and its mutants E::km, F::km and G::km were cultivated under chemolithoautotrophic conditions employing H_2_ plus CO_2_ as sources of energy and carbon and CO as an inducer of *cox* gene transcription (Fig. 2). Employing the protocol described in the methods section, the four CO dehydrogenases produced under these conditions were purified about 17-fold with an average yield of about 20 %. CO dehydrogenase amounted to approximately 6 % of the cytoplasmic proteins. The CO dehydrogenases showed the same mobilities on native PAGE, and their purity was apparent from a single protein band (Fig. 4a). Activity staining indicated the absence of CO-oxidizing activity in the CO dehydrogenases from the mutants E::km and F::km (Fig. 4b, lanes 2 and 3), whereas the enzymes from wild-type bacteria and the mutant G::km both displayed significant activities (Fig. 4b lanes 1 and 4). These results are corroborated by specific activities of the purified CO dehydrogenases (μmol CO oxidized mg^−1^ min^−1^) from wild-type bacteria and the mutants E::km, F::km and G::km of 8.529, 0.036, 0.136, or 4.360, respectively. The four CO dehydrogenases contained the required stoichiometric amounts of Mo and the FAD cofactor (Table 1).

**Table 1:** Analysis of CO dehydrogenases for FAD, metals and sulfane sulfur

Source of CO dehydrogenase	Wild type (CO)	Wild type (H_2_)	E::km (H_2_)	F::km (H_2_)	G::km (H_2_)
FAD	1.93 ± 0.08	1.98 ± 0.03	2.15 ± 0.06	1.92 ± 0.06	1.79 ± 0.04
Mo	1.85 ± 0.07	1.73 ± 0.10	1.95 ± 0.05	1.85 ± 0.03	1.91 ±0.08
Cu	1.62 ± 0.05	1.47 ± 0.03	0.00 ± 0.00	0.043 ± 0.00	1.48 ± 0.08
‘S’	1.93 ± 0.05	1.53 ± 0.01	2.0 ± 0.020	1.40 ± 0.13	2.17 ± 0.08
Zn	0.17 ± 0.01	0.22 ± 0.00	0.22 ± 0.00	0.076 ± 0.00	0.31 ± 0.01

Purified enzymes were obtained from *O. carboxidovorans* OM5 and its mutants E::km, F::km and G::km. Bacteria were cultivated under chemolithoautotrophic conditions employing a gas atmosphere of (v/v) 45 % CO, 5 % CO_2_, and 50 % air (referred to as “CO”) or 30 % H_2_, 5 % CO_2_, 30 % CO, and 35 % air (referred to as “H_2_”). All figures are in mol per mol of CO dehydrogenase determined from the absorption at 450 nm. FAD was estimated spectrophotometrically, metals by atomic absorption spectroscopy, and cyanolyzable sulfur (‘S’, sulfane sulfur) through cyanolysis. For details see “Materials and methods”. Standard deviations are based on at least three independent determinations

**Fig. 5:**
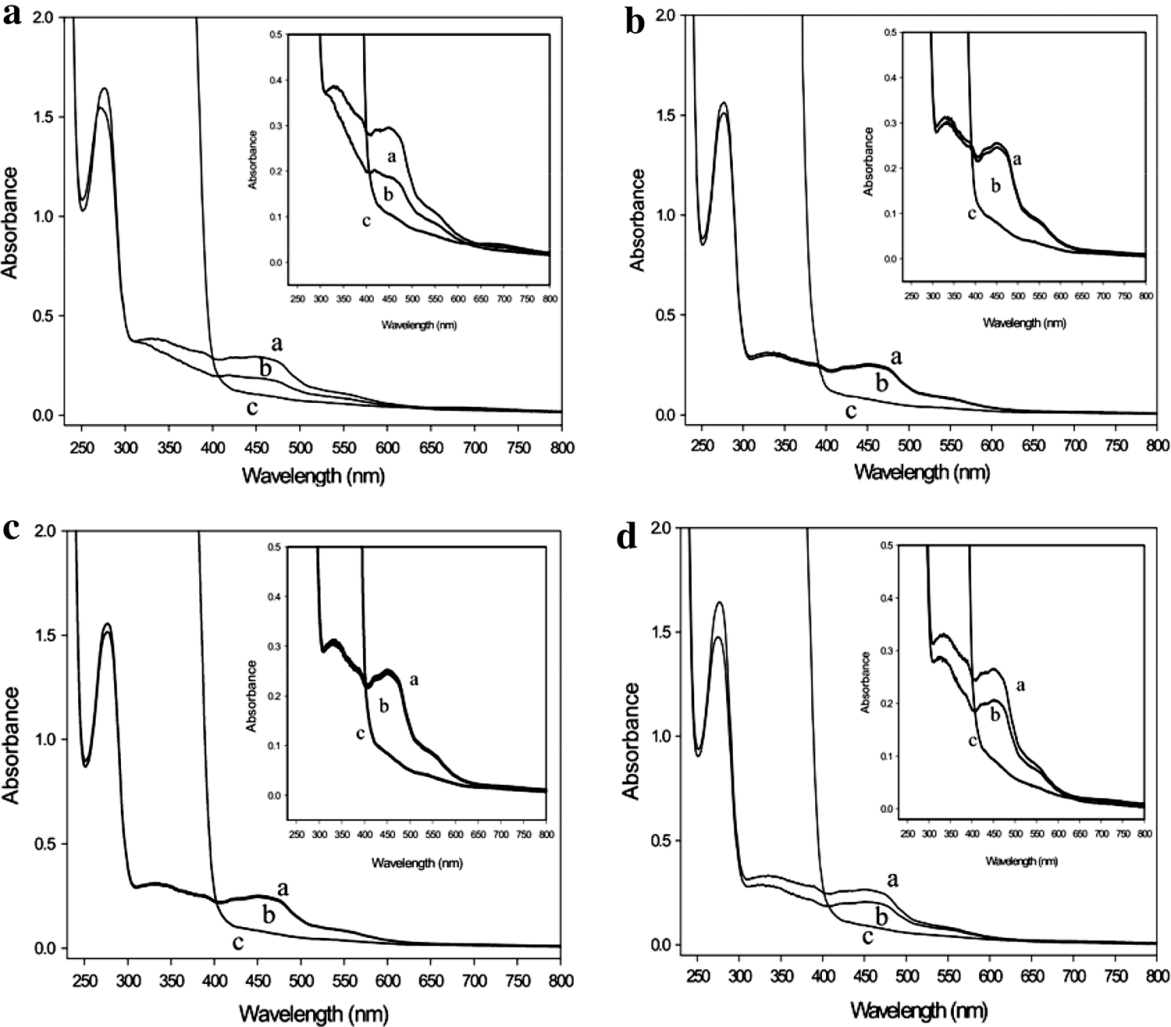
UV/VIS absorption spectra of CO dehydrogenases purified from wild type (**a**) or from the mutants in E::km (**b**), F::km (**c**) and G::km (**d**). Traces*: a*, air-oxidized; *b*, sparged with pure CO for 30 min; *c*, reduced with 650 µM dithionite under N_2_ for 4 min. The *insets* show the visible part of the spectra at greater detail

In addition, the UV/VIS absorption spectra were indistinguishable (Fig. 5) and showed absorption maxima indicative of FAD (~450 nm) and FeS (~550 nm). The observed absorption ratios of *A*_280_/*A*_450_ = 6.1 and *A*_450_/*A*_550_ = 3.1 indicate an iron–sulfur to flavin ratio of 4:1 present in wild-type CO dehydrogenase as well as in the enzymes from the mutants. The iron contents of the CO dehydrogenases from wild-type bacteria, E::km or F::km were (mol Fe per mol of enzyme) 7.67 ± 0.5, 7.67 ± 0.5, and 7.57 ± 0.5, which indicates that CoxE and CoxF are not involved in the incorporation of iron–sulfur clusters. The absorption maxima of FAD and the iron–sulfur centers of all four CO dehydrogenases were bleached by sodium dithionite (Fig. 5). Solely the enzymes from E::km and F::km were not bleached by CO (Fig. 5b, c) which indicates a defect in the catalytic site. The functionalities calculated from the spectra in Fig. 5 of the CO dehydrogenases from wild-type bacteria and the mutants E::km, F::km and G::km were 57, <0.1, <0.1, and 47 %, respectively. Obviously, the preparations of catalytically active CO dehydrogenase contain about 50 % of non-functional forms of the enzyme which has been recognized before [12]. These data show a specific involvement of the genes *coxE* and *coxF* in the maturation of a form of CO dehydrogenase which is already folded to the regular 3-D structure and has received molybdenum, the flavin cofactor and the iron–sulfur centers. Therefore, the defect in catalytic activity must be located in the bimetallic cluster.

Constituents and reactivity of the molybdenum site of the CO dehydrogenases from the mutants E::km, F::km and G::km.

In agreement with their ability to oxidize CO, the CO dehydrogenases from wild-type *O. carboxidovorans* cultivated with CO plus CO_2_ or with H_2_ plus CO_2_ in the presence of CO as an inducer of *cox* gene expression exhibited similar contents of Mo, Cu, and sulfane sulfur which was applied to the enzyme from the G::km mutant as well (Table 1). In contrast, copper was completely absent from the CO dehydrogenases of the mutants E::km and F::km, although they were complete in Mo and revealed stoichiometric amounts of sulfane sulfur (Table 1). Contaminating Zn was present in all preparations (Table 1).

**Fig. 6:**
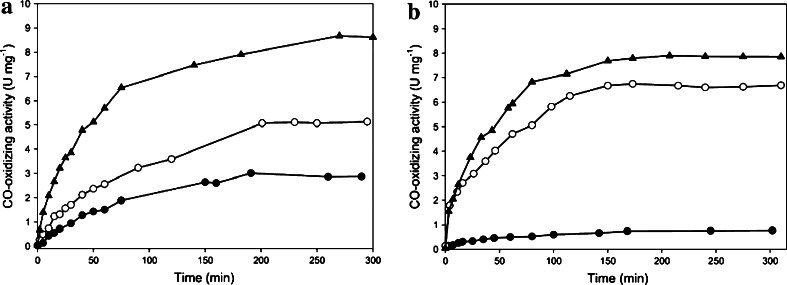
CO dehydrogenases from the mutants in *coxE* (**a**) or *coxF* (**b**) were treated with Cu^1+^-(thiourea)_3_ (*filled circle*) or with sodium sulfide and sodium dithionite first followed by Cu^1+^-(thiourea)_3_ (*open circle*). For the removal of Cu and cyanolyzable sulfur, CO dehydrogenases were incubated with potassium cyanide and then treated with sulfide/dithionite and copper as above (*filled triangle*). For details see the “Methods” section

When the CO dehydrogenases from the mutants E::km or F::km were treated with potassium cyanide to establish a [MoO_3_]-ion and then sulfurated followed by the introduction of Cu^1+^, significant CO dehydrogenase activities were obtained (Fig. 6; Table 2). Treatment of the two as isolated enzymes with Cu^1+^-(thiourea)_3_ reconstituted the CO-oxidizing activity to only 14 % (F::km) or 35 % (E::km) which was much lower than achieved by reconstitution of the [MoO_3_]-enzyme (set 100 %) or expected from the sulfane sulfur contents (Fig. 6; Table 2). The observation that obviously only a small fraction of the sulfane sulfur could bind Cu^1+^ identifies a separate fraction of sulfur which is reactive with potassium cyanide but cannot integrate Cu^1+^ into the active site. Although the two enzymes contained stoichiometric amounts (E::km) or nearly stoichiometric amounts (F::km) of cyanolyzable sulfur (Table 2), treatment with sodium sulfide plus sodium dithionite was necessary to achieve the reconstitution of substantial enzyme activity through the addition of copper (Fig. 6; Table 2). The treatment with sodium sulfide plus sodium dithionite did not increase the total cyanolyzable sulfur contents of the enzymes before the addition of Cu^1+^ which points to reactions at the Mo ion that are capable of improving the competence for Cu binding and activation (Table 2). Nevertheless, such treatment did not activate CO dehydrogenase to the same level as that obtained after reconstitution of the [MoO_3_]-enzyme with sulfide/dithionite/copper (Fig. 6; Table 2). It was also peculiar that the contents of cyanolyzable sulfur in the CO dehydrogenases from E::km and F::km dropped by up to 45 % upon exposure to Cu, whereas cyanide-treated and resulfurated enzymes kept their full complement of ‘S’ after the addition of Cu (Table 2). This might be taken as an indication of different forms of cyanolyzable sulfur, Mo-SH which allows restoration of activity through the addition of Cu^1+^ and another sulfur (presumably Mo-S-SH) which is of the wrong kind in this respect. The increased Cu contents of CO dehydrogenases treated with that metal of more than 2 Cu per mol of enzyme indicate the presence of so far unidentified Cu-binding sites in addition to the active site (Table 2). The additional Cu-binding sites might not be true sites but artifactual sites that have no physiological importance. Cu treatment is known to irreversibly damage the activity of other enzymes [53].

**Table 2:** Sulfane sulfur and copper in purified CO dehydrogenases which had received different treatments. E::km and F::km are the *O. carboxidovorans* mutants in *coxE* or *coxF*, respectively

Source of CODH	Treatment	S before (left) and after (right) the addition of Cu		Cu	Activity (μmol CO min^−1^ mg^−1^)
Wild type	A	1.95 ± 0.09	1.92 ± 0.11	8.07 ± 0.34	8.350
E::km	A	1.72 ± 0.16	1.98 ± 0.15	7.74 ± 0.22	8.617
	B	1.93 ± 0.13	0.87 ± 0.09	7.53 ± 0.63	5.113
	C	2.00 ± 0.02	1.12 ± 0.04	13.90 ± 0.38	3.016
F::km	A	1.47 ± 0.11	1.46 ± 0.26	8.85 ± 0.10	7.890
	B	1.58 ± 0.08	1.05 ± 0.11	8.40 ± 0.05	6.750
	C	1.40 ± 0.13	1.02 ± 0.35	11.60 ± 0.08	1.116

Treatments of CO dehydrogenases (5 mg ml^−1^): A, for the removal of Cu and sulfane sulfur, anoxic enzymes were incubated with 5 mM KCN for 24 h and then treated with 5 mM sodium sulfide plus 5 mM sodium dithionite first followed by 150 µM Cu^1+^(thiourea)_3_; B, as isolated enzyme was treated with sodium sulfide and sodium dithionite and then with Cu^1+^(thiourea)_3_; C, as isolated enzyme was treated with Cu^1+^-(thiourea)_3_. The sulfide/dithionite assays were incubated in the dark at 37 °C for 10 h, and the Cu^1+^-(thiourea)_3_- assays at 37 °C for 2–4 h in the dark. After each treatment the enzyme solution was gel filtered on PD10 ready-to-use columns (Sephadex G25, GE Healthcare, Little Chalfont, UK). All figures are in mol per mol of CO dehydrogenase. Refer to Table 1, Fig. 6 and the “Methods” section for further details

### Mo-EPR of the CO dehydrogenases from the mutants E::km, F::km and G::km

**Fig. 7:**
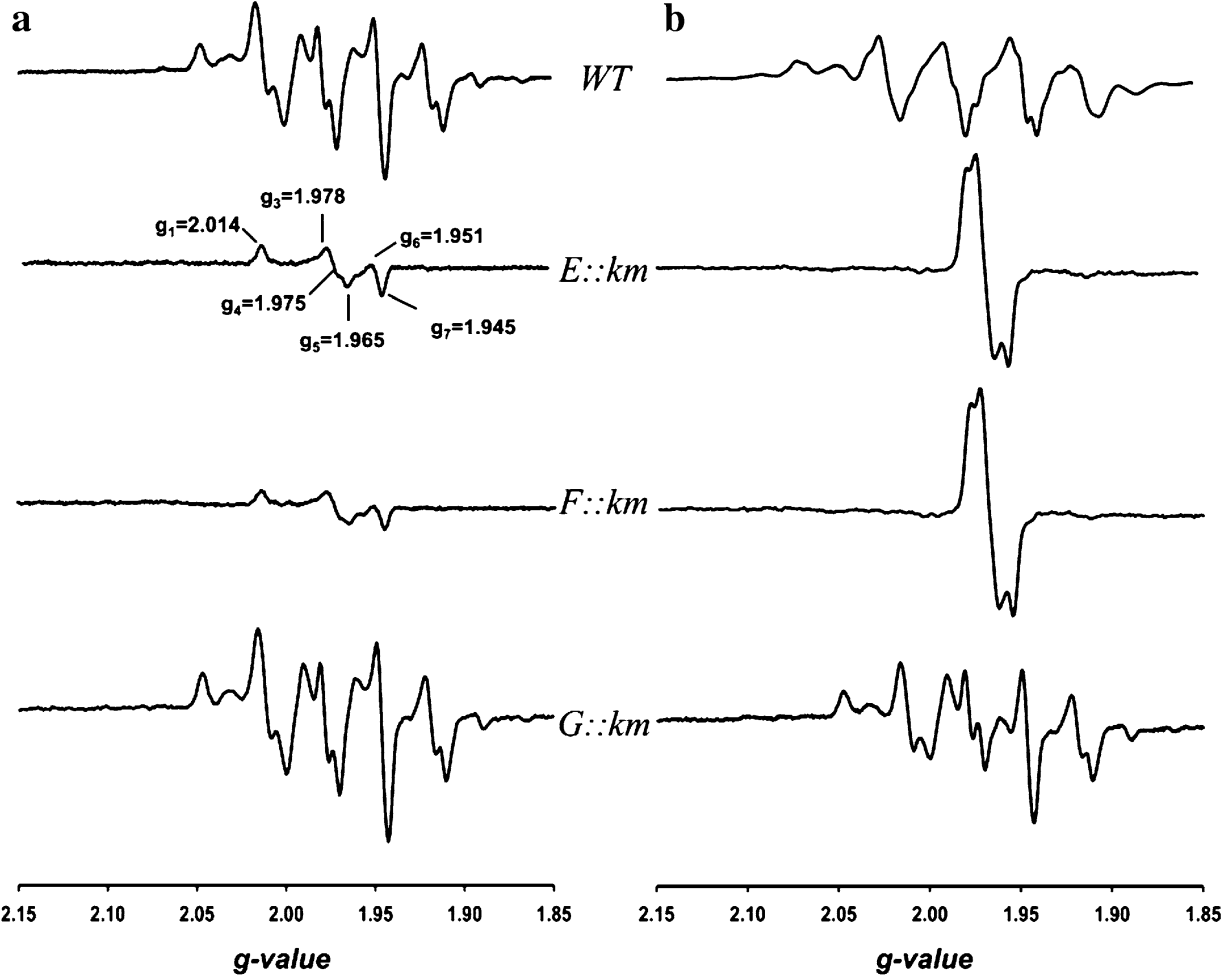
Mo-EPR of CO dehydrogenases (12 mg ml^−1^; 50 mM HEPES, pH 7.2) from *O. carboxidovorans* wild type and the mutants E::km, F::km, or G::km (from top to bottom). The enzymes were exposed to CO (**a**) or treated with 5 mM sodium dithionite (**b**). Spectra were recorded at 120 K at a microwave frequency, modulation amplitude, and microwave power of 9.47 GHz, 1 mT and 10 mW, respectively

In the presence of CO, the CO dehydrogenase preparations from wild-type *O. carboxidovorans* or the mutant G::km revealed the complex Mo(V)-EPR spectrum characteristic of the catalytically competent enzyme (Fig. 7a). The CO dehydrogenase preparations from the mutants E::km or F::km also showed paramagnetic Mo(V), however, the signal centered at *g* = 1.975 was not of the catalytically competent type and represented only 18 % (by spin integration) of the total Mo present (Fig. 7a), which agrees with the inability of both enzymes to oxidize CO (Fig. 4). Also sodium dithionite was able to produce a paramagnetic Mo(V) signal in the CO dehydrogenases prepared from wild-type bacteria and all mutants (Fig. 7b). This agrees with the presence of Mo in all enzyme preparations from the different sources shown in Table 1. The Mo-EPR spectra of the enzymes from E::km and F::km were indistinguishable referring to similar Mo sites (Fig. 7b). In accordance with chemical analysis (Table 1) the spectra corresponded to signals characteristic of Cu-deficient CO dehydrogenase [41], implying [MoO_3_]- and/or [MoO_2_S]-centers.

### Sulfur compounds as reporters of incomplete forms of the [CuSMoO_2_] cluster

**Fig. 8:**
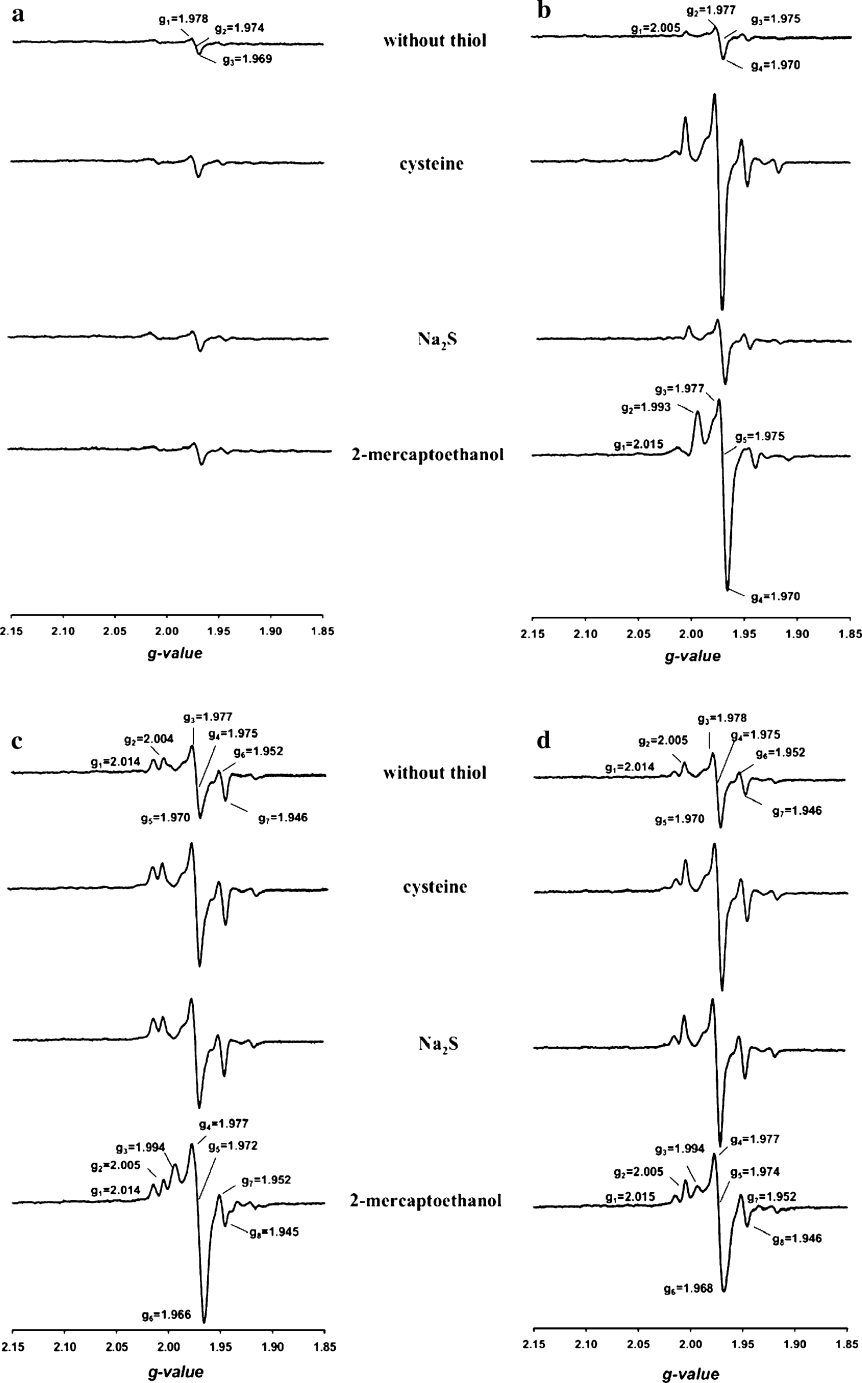
Mo-EPR of CO dehydrogenases in the presence of l-cysteine (15 mM), sodium sulfide (15 µM) or 2-mercaptoethanol (15 mM). **a** Enzyme from wild-type bacteria was treated with 5 mM potassium cyanide, small molecules were removed by gel filtration, and sulfur compounds were added as indicated. **b** Wild-type enzyme treated with 5 mM potassium cyanide was sulfurated (5 mM sodium sulfide plus 5 mM sodium dithionite) and, after gel filtration, supplied with the indicated sulfur compounds. The enzymes from the mutant E::km (**c**) or F::km (**d**) in their as isolated state were supplied with the indicated sulfur compounds. All assays contained CO dehydrogenase (12 mg ml^−1^) in 50 mM HEPES (pH 7.2) sparged with pure N_2_. EPR spectra were recorded as detailed in the legend to Fig. 7

l-cysteine, 2-mercaptoethanol and sodium sulfide were examined with respect to their ability to generate and/or alter paramagnetic EPR signals from species of CO dehydrogenase modified in the [CuSMoO_2_] cluster (Fig. 8). Treatment of CO dehydrogenase with potassium cyanide is known to convert the [CuSMoO_2_] cluster into a [MoO_3_]-center, a reaction which abolishes the ability of the enzyme to oxidize CO [11, 41]. Without added sulfur compounds, the [MoO_3_]-CO dehydrogenase prepared by cyanolysis of the wild-type enzyme afforded an EPR spectrum devoid of signals (Fig. 8a, below 3 % paramagnetic Mo by spin integration) referring to a mostly diamagnetic Mo(VI) ion. In the presence of cysteine, sodium sulfide or 2-mercaptoethanol the spectrum remained silent (Fig. 8a) which indicates the absence of one-electron redox interactions of the sulfur compounds with the [Mo^VI^O_3_]-site. [MoO_2_S]-CO dehydrogenase was produced by treatment of the [MoO_3_]-enzyme with sodium sulfide plus sodium dithionite (Fig. 8b). Its weak paramagnetic signal centered at *g* = 1.975 (about 7 % of the total Mo) significantly increased in the presence of sulfur compounds, particularly cysteine (32 % of the total Mo) or 2-mercaptoethanol (26 % of the total Mo) (Fig. 8b). These data indicate that the SH group of the studied sulfur compounds can bind to the equatorial sulfur of [MoO_2_S] under formation of a [Mo(O_2_)-S–S-R] mixed disulfide which is reported by EPR. The CO dehydrogenases obtained from E::km (Fig. 8c) or F::km (Fig. 8d) showed paramagnetic Mo(V) EPR signals similar to that of the [MoO_2_S]-enzyme in the presence of Na_2_S (Fig. 8b). Furthermore, the signals significantly increased upon the addition of cysteine or 2-mercaptoethanol (Fig. 8c, d). These data exclude a Cys-388 persulfide in the CO dehydrogenases from the two mutants and rather refers to the coexistence of two different types of sulfurated Mo, i.e. [MoO_2_S] and [MoO_2_-S–S–H]. The proportions of Mo species in the CO dehydrogenases from E::km (first mention) or F::km (second mention) deduced from spin integration were 2 to 21 % or 3 to 6 % of [MoO_2_S], 23 or 18 % of [MoO_2_-S–S–H] and 56 to 75 % or 76 to 79 % of [MoO_3_]. The coexistence of these Mo species explains the different levels of activation obtained upon reconstitution (Fig. 6).

### A pleckstrin homology (PH) domain is predicted on CoxG

Conserved domain searches revealed on CoxG a hydrophobic ligand site of the SRPBCC (START/RHO_alpha_C/PITP/Bet_v1/CoxG/CalC) super family. For structural sequence alignments of CoxG the bioinformatics program T-Coffee (http://www.tcoffee.org/) was used. The T-coffee search exhibited 62 % overall similarity of CoxG to the human pleckstrin 2 domain with especially good scores in the following patches: M^1^ to M^3^, R^13^ to I^36^, V^48^ to P^54^, F^59^ to L^65^, I^104^ to K^110^, T^128^ to K^139^, E^155^ to A^168^, and V^205^ (Fig. 1, green boxes). Disruption of the *coxG* gene did not impair the ability of *O. carboxidovorans* to utilize CO as a substrate for chemolithoautotrophic growth, except that the generation time was markedly increased (Fig. 2c). In addition, the CO dehydrogenase from the G::km mutant was complete in cofactor composition (Table 1). Although CO dehydrogenase from G::km was catalytically active, its specific CO-oxidizing activity was only 51 % that of the wild-type enzyme. It has been established that CO dehydrogenase is synthesized in wild-type bacteria as a mix of fully active and inactive species [12]. The inactive ones are complete in Mo and FAD but deficient in cyanolyzable sulfur and/or copper to a different extent. Apparently, this also applies to the CO dehydrogenase expressed in the absence of CoxG as it was complete in cyanolyzable sulfur but deficient in copper and fairly high in Zn which is suspected to occupy the Cu-binding site. Whether CO dehydrogenase expressed in the absence of CoxG might be trapped in a partially active precursor state of the active site requires further research. Evidently, the CoxG protein is not involved in the assembly of the bimetallic cluster. Instead, it is reasonable to assume that the PH domain on CoxG plays a role in recruiting soluble CO dehydrogenase to the inner aspect of the cytoplasmic membrane thus enabling electron transfer from the CO dehydrogenase FAD cofactor to the quinone pool [54] of the respiratory chain [55]. This interpretation is corroborated by the absence of particulate CO dehydrogenase in extracts of the G::km mutant [20].

## Discussion

### Types of Mo sites appearing in the assembly of the CO dehydrogenase bimetallic site

It is now clear that the genes *coxD*, *coxE* and *coxF* accomplish specific functions in the posttranslational sulfuration of a [MoO_3_]-site and the subsequent introduction of copper yielding a functional [CuSMoO_2_] cluster in CO dehydrogenase. The process proceeds at the molybdenum cofactor (Moco), composed of trioxo-Mo(VI) coordinated by the ene-dithiolate of molybdopterin-cytosine-dinucleotide (MCD), which is buried about 17 Å below the solvent accessible surface of apo-CO dehydrogenase [10]. The latter is properly folded and complete in cofactor composition, as it contains the iron–sulfur centers and the flavin-adenine-dinucleotide (FAD) cofactor. This sheds another light on what is currently known of molybdoenzyme maturation in bacteria, where sulfuration of the Moco occurs outside of the apoenzyme with the subsequent insertion of a matured Moco into a folded apoenzyme assisted by the dimeric protein XdhC or homologues thereof [25].

The mutual requirement of CoxE and CoxF for their individual translation led to the absence of both proteins in E::km and F::km (Fig. 3). As a consequence, the mutant F::km had the E::km phenotype, and the CO dehydrogenases from both mutants had similar properties. Enzyme preparations were inhomogeneous as they contained three types of metal sites in the quantitative order [Mo^VI^(=O)_2_OH_(2)_] > [Mo^V^(=O)OH_(2)_SSH] > [Mo^VI^(=O)OH_(2)_SH]. Of these, only for the persulfide a paramagnetic Mo(V) signal could be obtained (Fig. 8). In the oxidized state [MoO_2_S]-CO dehydrogenase showed only a weak paramagnetic EPR signal at 120 K (7 % of the total Mo) referring to a high proportion of diamagnetic Mo(VI) (Fig. 8b, trace *without thiol*). The presence of l-cysteine or 2-mercaptoethanol clearly increased the paramagnetic Mo(V)-signal (32 and 26 % of the total Mo, respectively) indicating a reduction by one electron (Fig. 8b, traces *cysteine* or *2*-*mercaptoethanol*). Prerequisite for such a reduction would be an interaction of the SH group with the Mo ion. Since the [MoO_3_] enzyme did not elicit a paramagnetic Mo(V) signal in the presence of cysteine and 2-mercaptoethanol (Fig. 8a) we conclude that a sulfur substituent at the Mo is required to enable the SH group of cysteine or 2-mercaptoethanol to react. The most likely reaction between two SH groups is the formation of a persulfide. The [Mo^VI^(=O)OH_(2)_SH] is considered as the biosynthetically relevant species. Variations in metal site composition also appear in CO dehydrogenase from wild-type *O. carboxidovorans* and have been ascribed to biosynthetic precursors of the bimetallic cluster [12, 41]. To address the relatively high proportion of [Mo^VI^(=O)_2_OH_(2)_], the XdhC motif at the N-terminus of CoxF is of importance (Fig. 1). It has been reported that in the absence of XdhC inactive xanthine dehydrogenase contained Moco, but the terminal sulfur ligand required for activity was missing [56]. The strong influence of oxygen supply suggested a role of XdhC in protecting the exchange of the sulfur ligand of Moco against an oxygen atom, and it was assumed that XdhC particularly stabilizes the sulfurated form of Moco before the insertion into its target protein [56]. Taking this analogy, it can be assumed that CoxF protects the sulfurated Mo species in CO dehydrogenase from oxidation. Therefore, the absence of CoxF in the mutants E::km and F::km (Fig. 3) is likely to increase a spontaneous oxidative removal of the sulfur ligand from [Mo(=O)OH_(2)_SH] resulting in the formation of [Mo(=O)_2_OH_(2)_]. The loss of sulfur seems to be an undirected, statistic process. As a result, the mutants in *coxE* and *coxF* contain apoenzymes with [Mo(=O)_2_OH_(2)_] centers to different extents and slightly differ in the in vitro activation (Fig. 6). The presence of [Mo(=O)OH_(2)_SH] and [Mo(=O)OH_(2)_SSH] in the CO dehydrogenases from E::km and F::km indicates that the corresponding genes operate in the introduction of copper into a sulfurated molybdenum site.

### Prediction of CoxD, CoxE and CoxF as DEAD-box proteins and anticipated functions

Members of the DEAD-box family of RNA helicases use ATP to rearrange RNA and RNA–protein structures [45, 57, 58]. They consist of a helicase core containing 12 conserved motifs involved in ATP binding, RNA binding and ATP hydrolysis [59]. Five homologues appear on CoxD (Ia, I, IV, II, V), two on CoxE (Ib, III) and two on CoxF (III, IV) (Fig. 1) with the corresponding sequences: Ia, P^21^DRDLA^26^; I, G^43^EAGVGKT^50^; IV, L^64^IR^66^; II, D^134^EVD^137^; V, A^204^RIIT^208^ (CoxD); Ib, T^54^PSR^57^; III, S^91^AT^93^ (CoxE), S^31^LT^33^ and S^178^GT^180^ (CoxF), IV, L^224^TV^226^ (CoxF). The motiv III is duplicated on CoxF (Fig. 1). Because of the presence of these DEAD-box motifs, it can be envisioned that CoxD can accomplish functions of a RNA-helicase engaged in translation, such as eIF4A from *Saccharomyces cerevisiae* [60]. CoxE and CoxF carry only a small number of DEAD-box motifs which is in contrast to CoxD (Fig. 1). On the other hand, the motifs Ib and III on CoxE and the motif III on CoxF are special because they are not present on CoxD. This might point to some sort of cooperation in translation between CoxD, CoxE and CoxF. Indeed, CoxD can hydrolyse MgATP [44], and RNA-helicase functions of the three Cox proteins would explain their mutual requirement for translation (Fig. 3). Furthermore, the insertion of a kanamycin resistance cassette might exert an effect on the stability of the mRNA in the mutants. The mutation in *coxF* could influence mRNA folding, particularly increase the stability of the *coxD* message in F::km and/or might enhance the accessibility of the ribosome-binding site. As a result, higher expression levels of CoxD can be achieved (Fig. 3a).

### Metal cluster maturation

**Fig. 9:**
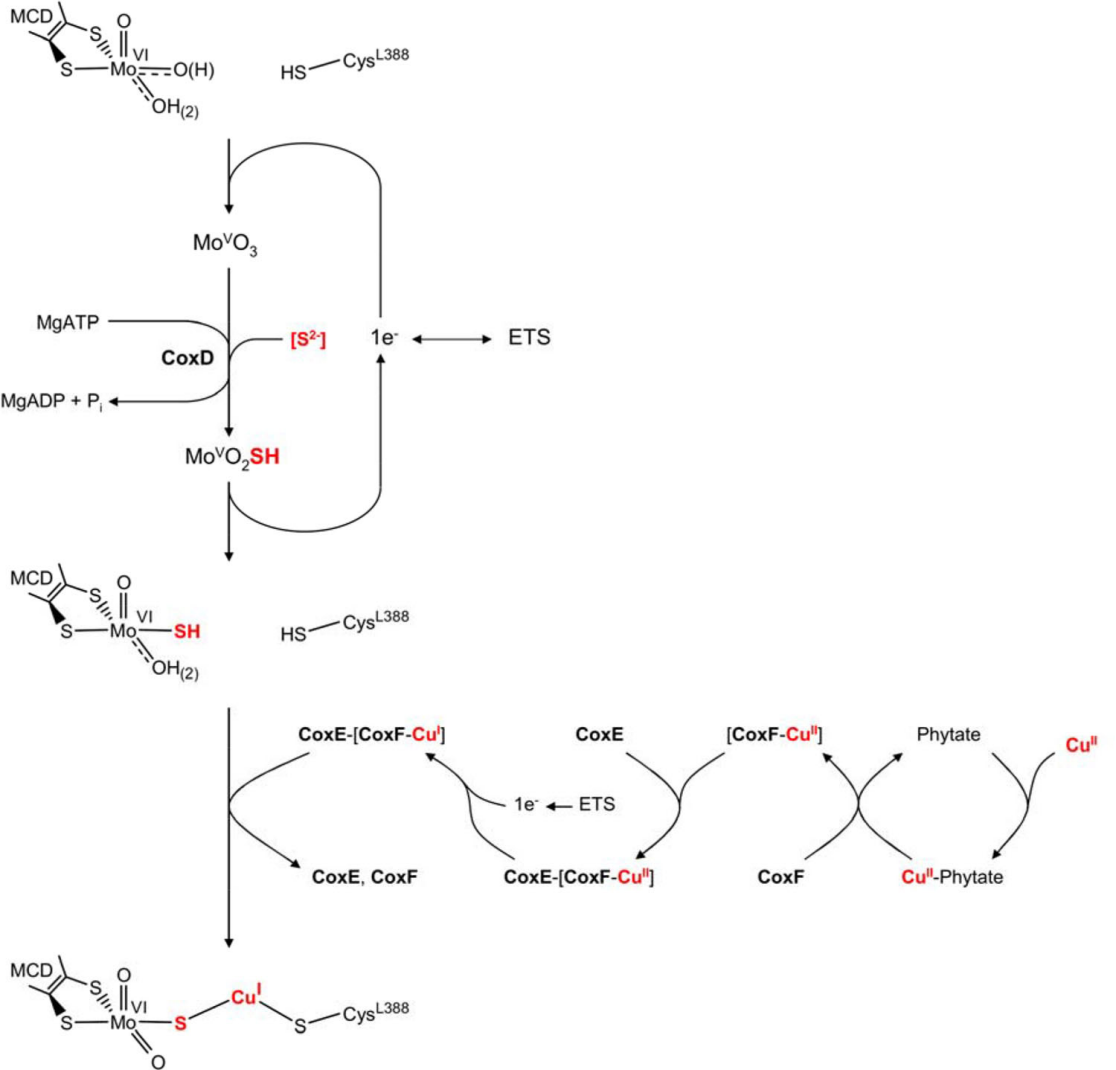
Model showing proposed functions of the proteins CoxD, CoxE and CoxF in the assembly of the Mo- and Cu-containing cluster in the active site of folded apo-CO dehydrogenase. CoxD is an AAA+-ATPase chaperone required for the sulfuration of the trioxo-Mo ion. CoxF, which is attached to the cytoplasmic membrane through complex formation with the von Willebrand protein CoxE, introduces a Cu^1+^-ion resulting in a catalytically competent [CuSMoO_2_] center. CoxF employs suspected phytase activity for the release of Cu^2+^ attached to phytate and a putative Cu-binding motif to escort the metal ion. The respiratory electron transport system (ETS) supplies electrons for the generation of Cu^1+^ from Cu^2+^. See the text for further explanations

Solely the mutant G::km synthesized a fully active enzyme completely in sulfur and copper (Table 1) suggesting a [Mo(=O)OH–S–Cu–S-Cys] cluster which indicates that CO dehydrogenase biosynthesis is complete prior to the action of *coxG.* The posttranslational assembly of the [Mo(=O)OH-S-Cu–S-Cys] cluster in the active site of CO dehydrogenase is a complex and highly ordered process which involves the introduction of sulfur and copper into a [Mo^VI^(=O)_2_OH_(2)_] site (Fig. 9). It represents the final step of cluster maturation, resulting in a catalytically active enzyme. We have shown herein that the biosynthesis of the Mo/Cu cluster involves the functions of the genes *coxE* and *coxF*, in addition to *coxD* [29, 44]. Cluster biosynthesis starts with the one-electron reduction of [Mo^VI^(=O)_2_OH_(2)_] to [Mo^V^(=O)_2_OH_(2)_] by an unknown mechanism (Fig. 9). However, since CoxD is a membrane protein [44], the shuttle between Mo^VI^ and Mo^V^ can be imagined to involve the electron transport system (ETS). The next step is the MgATP-dependent sulfuration of [Mo^V^(=O)_2_OH_(2)_] to [Mo^V^(=O)OH_(2)_SH] which involves the AAA-ATPase chaperone CoxD [29, 44]. It is not known whether CoxD itself acts as a sulfurtransferase and what the actual sulfur source is. However, cysteine or thiosulfate is sulfur donor which must be considered [25, 56, 61]. Our data along with a previous report [62] show that thiol compounds much larger than CO (e.g. l-cysteine or 2-mercaptoethanol) can freely travel through the substrate channel leading to the CO dehydrogenase active site. This challenges previous concepts involving chaperone function of CoxD on apo-CO dehydrogenase [29].

The in vivo oxidation state of Mo in [Mo(=O)OH_(2)_SH] must be +VI to enable a transfer of Cu^1+^. This is suggested by the conditions of chemical reconstitution and EPR described previously [41] as well as from the experiments shown in Figs. 6 and 8. The incorporation of Cu^1+^ involves CoxE and CoxF which is indicated by the absence of Cu in the CO dehydrogenases from E::km and F::km (Table 1). Although the exact functions of CoxE and CoxF must await studies at the protein level, sequence information suggests the Cu acquisition shunt depicted in Fig. 9. CoxF reveals signatures of a potential histidine acid phytase (HAPhy, R^266^HGQRQS, RHGXRXP) and a suspected Cu-binding site (M^107^CPSHGTM; [47] (Fig. 1). These could enable CoxF to release phytate-bound Cu through the hydrolysis of phosphate monoesters with the subsequent transfer to the Cu-binding site. Complex formation of soluble CoxF(Cu) and membrane-bound CoxE through their RGD motif and the VWA domain establishes access to the electron transport system (ETS) for the reduction of Cu^2+^ to Cu^1+^ (Fig. 9). Finally, Cu^1+^ transfer from its escorting protein CoxF to [Mo^VI^(=O)OH_(2)_SH] in apo-CO dehydrogenase results in the formation of a complete and functional bimetallic cluster (Fig. 9). CoxE is an integrin with a single metal ion-dependent adhesion site (MIDAS) located in its ligand-binding site.

CoxE is likely to form an intermediate ternary complex with a cation and CoxF to regulate ligand binding (Fig. 1). The types of cations involved in this process, and particularly any role of Cu, are currently not known. Characterizing CoxD, CoxE and CoxF at the protein level and identifying the missing factors in the maturation of the bimetallic site of CO dehydrogenase are important challenges for the future.

## Abbreviations

AAS: Atomic absorption spectroscopy
EPR: Electron paramagnetic resonance
FAD: Flavin adenine dinucleotide
MCD: Molybdopterin cytosine dinucleotide
MIDAS: Metal ion-dependent adhesion site
OD: Optical density
PAGE: Polyacrylamide gel electrophoresis
PHP: Pleckstrin homology
SDS: Sodium dodecyl sulfate
UV/VIS: Ultraviolet/visible
VWA: Von willebrand factor A
XdhC: Xanthine dehydrogenase C
